# Exploring the Role of Nursing Ethics in Shaping Information Security Culture: A Normative and Care Ethics Perspective

**DOI:** 10.1155/jonm/9993858

**Published:** 2025-12-18

**Authors:** Samanta Mikuletič, Simon Vrhovec, Boštjan Žvanut

**Affiliations:** ^1^ Primary Health Care Centre Ilirska Bistrica, Gregorčičeva Cesta 8, Ilirska Bistrica, 6250, Slovenia; ^2^ Faculty of Criminal Justice and Security, University of Maribor, Kotnikova Ulica 8, Ljubljana, 1000, Slovenia, um.si; ^3^ Faculty of Health Sciences, University of Primorska, Polje 42, Izola, 6310, Slovenia, upr.si

**Keywords:** ethics of care, information security culture, normative ethics, patient privacy

## Abstract

**Aim:**

An appropriate information security culture (ISC) is essential for protecting patient privacy and safety. This study aims to explore the role of normative and care ethics in shaping ISC and to propose a novel nursing ethics ISC framework.

**Design:**

The study employed a qualitative exploratory design based on semistructured interviews, including nurses and IT experts. Realistic scenarios involving two fictional registered nurses (RNs) were used to mitigate potential biases and encourage open and honest discussion on sensitive topics. Thematic analysis was used to derive insights from different participant perspectives.

**Methods:**

Semistructured interviews were conducted between 23 November 2019 and 22 June 2020 with 13 nurses and four IT experts working in clinical settings in Slovenia. A thematic analysis was conducted.

**Results:**

Our in‐depth analysis shows that ISC is shaped by both normative and ethics of care perspectives, supporting the proposed nursing ethics ISC framework. Normative factors included procedural security countermeasures, direct superior’s commitment to information security, information security knowledge, and security monitoring. Additionally, we identified two factors related to ethics of care, namely, attention to patient dignity, and attentiveness to and alleviation of patient vulnerability.

**Conclusion:**

This is one of the first studies to provide support for including both normative and ethics of care perspectives when studying ISC in the context of nursing ethics. The identified ISC factors also provide a reference for future research endeavors, such as the operationalization of instruments for measuring these concepts in nursing.

## 1. Introduction

Information security and privacy are becoming increasingly important due to the rapid digitization of health records and the growing need for their use and exchange. This raises various organizational, technical, legal, and last but not least ethical challenges [1, 2]. Protecting health data is particularly challenging [2] due to “potentially existential threats” and a growing number of information security breaches [3].

Health data are among the most sensitive and confidential personal information, the unauthorized disclosure of which leads to significant legal and financial consequences [4, 5]. Apart from these consequences, ethical and moral responsibilities are also of utmost importance. The improper disclosure or misuse of health data can cause serious damage to patients’ reputation, such as discrimination, stigmatization, loss of insurance, and/or loss of employment [6].

Various technical means are available to protect health data; however, despite their importance, they are not sufficient on their own [7]. For example, compliance with information security policies (ISPs) is important to prevent data breaches [8, 9]. During a typical nursing care process, up to 400 individuals may have access to a patient’s sensitive health data. Since nurses represent a large portion of these, information security forms an important part of their daily work practices [10]. Adequately trained and reliable employees who adhere to organizations’ information security procedures and moral norms are, therefore, critical to prevent information security breaches. The ICN Code of Ethics [11] directly addresses nurses’ attitudes toward information security.

To effectively address the aforementioned risks, information security culture (ISC) is of paramount importance to organizations [12]. Healthcare and other organizations can effectively address this alarming problem by cultivating their ISC [13], as it helps to develop appropriate security behavior among their employees’ and support the achievement of their organizational goals.

ISC is an important element of organizational culture and refers to all forms of efforts aimed at mitigating the risk of information security breaches and incidents [12, 14]. ISC, as part of organizational culture, can be seen as a set of organizational characteristics relevant to ensuring the security, availability, and integrity of data, that is, assumptions about what is (not) acceptable in terms of information security [15]. Similar to organizational culture, ISC may take several years to be adequately implemented and cultivated [16]. There are several definitions of ISC in literature; however, there seems to be some confusion regarding the definition of ISC, ISC factors, and ISC dimensions. First, there are two widely used definitions of ISC—a broader one and a narrower one. Under the broader definition, ISC can be summarized as “*the way we do things around here*” [17]. The narrower definition describes ISC as deeply rooted and less observable and defines information security climate as a visible manifestation of ISC, which includes perceptions about organizational policies, practices, and procedures [18]. Second, there seems to be a lack of consensus on ISC factors/dimensions [19–21]. Moreover, there are different approaches to the conceptualization and operationalization of ISC, some of them based on unidimensional [22–24] and others on multidimensional [25] constructs. Some authors tend to mix the two concepts, which leads to further confusion. Third, ISC is operationalized in different ways. While there are a few studies which operationalize ISC as a stand‐alone construct [13, 24, 26–33], most of them operationalize it as a composite construct. These studies construct ISC from ISC factors [15, 25, 34–36], ISC dimensions [37–42], or ISC manifestations (i.e., information security climate) [18, 43, 44]. Fourth, there seems to be no consensus regarding ISC dimensions, as some studies consider dimensions as factors and vice versa. For example, Chen et al. [27] list security education, training, and awareness (SETA) programs and security monitoring as ISC factors, while Nasir et al. [40] consider them as ISC dimensions. All these points contribute to an unclear theoretical underpinning of ISC.

Further insights can be obtained from literature. ISC is an important concept in healthcare as several ISC studies have focused exclusively on this domain [13, 18, 26, 32, 36, 42–44] or have included healthcare institutions as part of a broader study [24, 25, 29, 38, 41, 45, 46]. Despite the fact that several studies in different fields have emphasized the importance of ethics in information security [13, 15, 18, 25, 26, 28, 30, 33–36, 39, 41], only two studies (i.e., [15, 46]) examined the relationship between ethics and ISC. Recently, studies focused on ethical use of artificial intelligence (AI) in nursing with some relevance to ISC (e.g., use of AI and privacy and training) [47–49]. However, none of these studies has explored ISC through the nursing ethics perspective—marking a critical gap that this research seeks to address.

The common denominator of studies found in the literature is that ISC factors and dimensions are grounded in the organizational and managerial aspects of ISC, which align with key approaches within normative ethics [50–52]. Normative ethics deals with the principles that determine what is morally right or wrong and involves the development of moral standards guiding human behavior, social institutions, and ways of living [51, 53]. In the literature, there are many approaches to normative ethics, most common are deontology, consequentialism, and virtue ethics [52]. However, in the context of nursing, ISC may need to extend beyond the confines of normative ethics to include an ethical element unique to nursing, namely, the ethics of care. Ethics of care (also referred to as care ethics) emphasizes the moral significance of caring relationships and acknowledges the relational and vulnerable nature of individuals [54]. Ethics of care has already been identified as a potential guiding philosophical framework in information security [55]. Normative and care ethics are cornerstones of nursing ethics, prescribing how practice should be conducted in nursing contexts while considering the uniqueness of individuals and their situations. They may both contribute to understanding the emergence of ISC within the context of nursing. However, previous studies have not yet considered this perspective in the conceptualization of ISC, as the field of ISC in nursing has not yet received adequate attention.

The objective of this study was to identify the key ISC factors and develop a novel ISC framework focusing on nursing ethics. This is one of the first studies to explore ISC emergence in nursing from both normative and care ethics perspectives, given that ethics of care is a fundamental aspect of nursing practice while also being closely intertwined with information security. Understanding these perspectives is essential for nurse managers and healthcare leadership since fostering a strong ISC—grounded in ethical nursing practice—is critical for protecting patient data, ensuring compliance, and promoting trust within healthcare teams.

## 2. Methods

### 2.1. Design

A qualitative exploratory design based on semistructured interviews was conducted. The qualitative research method of thematic analysis was used for identifying and interpreting themes emerging from the participants’ views.

### 2.2. Participants and Settings

Slovenian nursing staff members and IT experts were recruited between 23 November 2019 and 22 June 2020 through purposive sampling. To capture a more comprehensive understanding of ISC in nursing, we also included IT experts who work closely with this population. This heterogeneous sample enabled us to observe ISC from the perspectives of both nursing professionals and those directly involved in supporting their work. Our sample consisted of a total of 17 participants. Their age ranged from 26 to 60 years (*M = *37.2 and SD = 9.6). Ten participants were female and seven were male. Participants were employed at all levels of the healthcare system: four at the primary healthcare level, seven at the secondary level, four at both secondary and tertiary levels, and two at the tertiary level. A total of 13 nursing staff members participated, that is, 2 nursing technicians and 11 registered nurse (RN) (four BSc, six MSc, and one PhD), and four IT experts (two BSc, one MSc, and one PhD).

### 2.3. Interview Guide

A semistructured interview protocol was designed and subsequently piloted with two volunteers. The core of the interview protocol was two realistic scenarios and an open‐ended questionnaire, presented in Table 1, which served as a guide for conducting the interviews.

**Table 1:** Fictional scenarios and open‐ended questions.

Code	Item
Scenario 1	Please, imagine two RNs: Marina and Mathew. In order to perform their work activities, they need to access their patients’ data. If they wish, they can access the data of all patients, but they are not authorized to do so.
Q1.1	What do you think most influences Marina and Mathew in their decision not to access the data of patients for which they do not have authorization? [If needed: What else?]
Q1.2	What do you think most influences Marina and Mathew in their decision to access the data of patients for which they do not have authorization? [If needed: What else?]
Scenario 2	Please, imagine two RNs: Marina and Mathew. Marina forgot her password; in principle, she should contact the IT department for a reset, but she can also decide to ask her colleague Mathew to lend her his credentials.
Q2.1	What do you think influences Marina’s decision to borrow the password? [If needed: What else?]
Q2.2	What do you think influences Marina’s decision not to borrow the password [If needed: What else?]

Two typical information security breaches (i.e., unauthorized access to their patients’ data and password disclosure) were addressed in the interviews. These acts were selected because (1) they are among the most common information security breaches [3, 56] and (2) the participants of the study were familiar with them. The questions were phrased in the third person and projected onto the behavior of two fictional characters (Marina and Mathew, both RNs) so that the interviewees could talk openly about the behavior of these individuals. Using fictional scenarios helps address sensitive issues without compromising participants’ confidentiality or ethical standards, which is particularly relevant in the context of information security, where discussing actual breaches may be uncomfortable or legally sensitive [57].

### 2.4. Data Collection Procedure

The interviews were conducted face‐to‐face. Each interview began with a presentation of the context and ethical considerations of the study (see section Ethical considerations). The interviews took place at a time and location that suited the participants. Particular attention was paid to limiting distractions. Only the researcher and the interviewee were present during the interview. Interviews were tape‐recorded, transcribed verbatim, and checked for errors by a researcher who did not conduct the interviews. The duration of the interviews ranged from 15 to 30 min. Handwritten notes were taken during each interview to record emerging thoughts and ideas. These served as references for new questions. None of the interviewees refused to participate. Of the 17 interviews conducted, data saturation was reached at the 10th interview. To increase the quality and reliability of the study, seven additional interviews were conducted.

### 2.5. Ethical Considerations

The proposal was approved by the Commission for Scientific Research Work at the Faculty of Health Sciences at the University of Primorska on 11 November 2019. Prior to each interview, participants were familiarized with the purpose of the study, the procedure, its potential benefits/risks, details regarding participant confidentiality and recording of the interview, the right to refuse to be recorded, the voluntary nature of participation, and the right to withdraw at any time. Each participant signed the informed consent form for participation in the study and recording of the interview.

### 2.6. Data Analysis

A thematic analysis was conducted to identify, analyze, and report on the themes identified in the transcripts [58]. The Consolidated Criteria for Reporting Qualitative Research [59] was used as a guide. A naïve reading was conducted as a first step, followed by the coding process recommended by Chapman et al. [60]. Open‐ended descriptive coding was performed independently by two researchers, who organized the transcripts data into sets of codes representing short statements that could be associated with similar ideas or meanings. To reduce researcher subjectivity both researchers systematically documented the coding process. After open coding, both researchers compared and consolidated the identified codes and repeated the coding process. In the second step, the codes were grouped into themes according to their (dis)similarity and checked for completeness; potential relationships between themes were also identified. Throughout these first two steps, special care was taken to avoid simplistic analytical inferences from raw data to pre‐existing concepts and theories. In the final step, the identified themes were compared with existing ISC factors/dimensions and nursing ethics concepts relevant to this study. These themes were then grouped to develop a novel ISC framework, specifically focusing on nursing ethics. Atlas.ti 7 was used to manage, code, and analyze the transcripts.

## 3. Results

**Figure 1:**
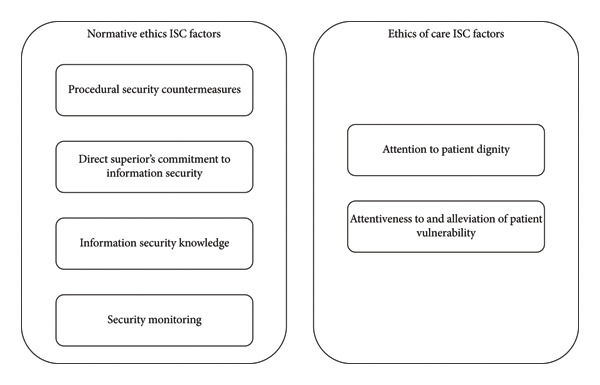
ISC factors identified within the novel nursing ethics ISC framework.

After identifying the emerging themes and comparing them with the published literature, we developed a novel nursing ethics ISC framework consisting of two groups of ISC factors: (1) normative ethics ISC factors and (2) ethics of care ISC factors. A visualization of the developed nursing ethics ISC framework is presented in Figure 1.

### 3.1. Normative ISC Factors

#### 3.1.1. Procedural Security Countermeasures

The interviewees reported several elements found in information security policies, referred to as procedural security countermeasures (e.g., automatic, logoff after a certain period of inactivity, access restrictions, and policies on the use of credentials). Analysis of their responses shows that they are aware of the importance of these countermeasures in preventing information security breaches, for example:

*‘If you fail to do that, the computer will automatically log you off after 120s.’* [Nurse 02, 27 years, female]

*‘In the hospital, there has basically always been a rule that you cannot access data on patients who have not been placed in your care. Yet these rules have often been violated.’* [Nurse 12, 30 years, male]

#### 3.1.2. Direct Superior’s Commitment to Information Security

The interviewees pointed out the importance of superior’s attitude to information security issues. All quotes related to this theme were addressed to the direct superior, i.e., manager to whom the employee reports directly. The interviewees emphasized the importance of the superior’s commitment to information security and particularly their enforcement of ISC policies.

*‘In some cases, even though my superior knows that I do not have the credentials, they advise me to borrow them from my colleagues.’* [Nurse 03, 29 years, female]

*‘They will probably borrow their credentials if their superior indirectly pressures them to take this action in order to continue their work.’* [IT Expert 02, 47 years, male]

*‘I am sure that he/she will not borrow his/her credentials if he/she receives the instruction from the superior not to do so under any circumstances.’* [IT Expert, 01, 46 years, male]

#### 3.1.3. Information Security Knowledge

Information security knowledge (e.g., knowledge in data protection and internal organizational acts) was identified in the responses of the interviewees as an important determinant of behavior directed to prevent information security breaches:

*‘The knowledge of data protection and the fact that it is not even necessary to do that [i.e., take this action] causes Marina and Matthew not to access patient data for which they are not authorized.’* [Nurse 09, 60 years, female]

*‘Mathew will never borrow his credentials if he is familiar with the organization’s internal acts and has attended a training where he has been made aware of the consequences of such action.’* [IT Expert 01, 46 years, male]

#### 3.1.4. Security Monitoring

According to the interviewees’ responses, various security monitoring mechanisms (e.g., collecting and analyzing audit logs for unauthorized security‐related activities) can effectively prevent information security breaches, for example:

*‘I would certainly not borrow my credentials as the computer records every activity and associates it with those credentials.’* [Nurse 08, 29 years, female]

*‘[…] awareness of the existence of an audit trail represents an additional motive to refrain from such actions (unauthorized data access).’* [IT Expert 01, 46 years, male]

### 3.2. Ethics of Care ISC Factors

From the participants’ responses, the following subthemes emerged as critical to protecting patient privacy and safety: (1) *attention to patient dignity* and (2) *attentiveness to and alleviation of patient vulnerability*. These were identified as ethics of care ISC factors since they can directly influence nurses’ information security behavior.

#### 3.2.1. Attention to Patient Dignity

Interviewees pointed out that information security breaches can severely affect patient dignity. They pointed out that the attention to patient dignity enhances their critical thinking in various situations where there is a risk of a security breach and, more importantly, it guides them in decision making whether to perform a certain behavior or not, for example:

*‘For example, if somebody accessed the information about why I am on sick leave, and if the diagnosis could lead to stigmatization, I (as a patient) would not feel good.’* [Nurse 08, 28 years, female]

*‘When you look at other patients’ information [i.e., patients who are not under my care], you are invading their privacy… and this is not right.’* [Nurse 13, 27 years, female]

*‘If one asks oneself, what would happen if this matter [i.e., the confidential information about the patient’s health condition] became public or if it reached the patient’s relatives?’* [Nurse 04, 45 years, female]

#### 3.2.2. Attentiveness to and Alleviation of Patient Vulnerability

It is evident in the responses of the interviewees that they are aware that the mere existence of health records in information systems makes patients vulnerable. The interviewees pointed out the importance of being attentive to behaviors that may increase this vulnerability (e.g., unnecessary access to patient records that may lead to disclosure of certain information), and they try to do their best to alleviate this vulnerability, e.g., by avoiding or not performing these behaviors. It appears that interviewees strive to mitigate the above vulnerability and are clearly mindful of the potential consequences of an information security breach:

*‘If they [i.e., Marina and Mathew] do not treat the patient at all, they have no right to look at the patient’s records because they are already abusing them by doing so*.*’* [Nurse 07, 47 years, female]

*‘We (nurses) should not access the data of those patients who are not in our care. This is crucial in order to prevent their misuse.’* [Nurse 08, 28 years, female]

*‘It is not right to access other people’s data […] The reason is curiosity […], they might also wish to sell this data, for example, to journalists.’* [Nurse 03, 28 years, female]

## 4. Discussion

Interviews with our respondents indicate that ISC in nursing encompasses organizational factors grounded in both normative ethics and the more recently introduced ethics of care perspectives. Our study expands the existing understanding of ISC by suggesting that, within nursing, ISC is shaped by these two complementary ethical approaches. The results of this study provide backing for the proposed nursing ethics ISC framework, suggesting a need for a more comprehensive view of ISC in the context of nursing. While our study is focused on nursing, the findings may also have broader relevance beyond this field.

Several normative ISC factors previously documented in the literature, such as procedural security countermeasures, security monitoring, and information security knowledge, were confirmed as significant by our interviewees. Interestingly, although nurses acknowledged the importance of information security knowledge, none of them highlighted information security knowledge sharing or SETA. This omission is surprising because nurses generally view knowledge sharing as essential for their professional growth [61]. This suggests that, at least among our respondents, information security knowledge and skills are not yet regarded as priorities for ongoing education or as critical components of knowledge sharing.

A further noteworthy divergence emerged regarding the ISC factor of direct superior’s commitment to information security. While [40] highlight the critical role of top management’s attitude toward information security, none of our interviewees mentioned top management but instead focused on direct superiors, such as head nurses, reflecting middle management influence. This aligns with earlier studies [18, 62] which show that immediate supervisors impact employee behavior more than top executives. This is especially relevant in nursing, where nurse leaders serve as role models [63], shifting ISC focus from strategic to operational decision‐making levels. Managers strongly influence their nursing teams [64, 65], making it essential that employees recognize their role in organizational information security [40].

The authors in [55] noted that ethics of care remains insufficiently integrated into moral philosophy and many areas of daily life. Given the scarcity of research on ISC in nursing, it is unsurprising that our findings show ISC has rarely been considered through the ethics of care lens, despite its acknowledged importance for information security [55] and security more broadly [66].

Fundamental principles of the ethics of care, that is, protecting human dignity and alleviating patient vulnerability [67, 68], are particularly relevant in clinical contexts, where patients risk losing dignity via breaches of information privacy [69]. Establishing an organizational culture that promotes patient dignity is, therefore, crucial [70]. Respect, a core aspect of dignity, encompasses self‐respect, respect for others, and respect for privacy and confidentiality [71]. From an information security standpoint, respecting patient privacy and confidentiality is especially vital. Duty‐based ethics mandate that nurses protect these rights [72], a stance also enforced legally [73]. Yet, going beyond legal requirements to actively respect patient dignity through safeguarding privacy and confidentiality contributes to their practical realization. Nurses’ moral courage is characterized by attentiveness to patient vulnerability [74]. In information security, this vulnerability extends beyond illness or suffering to the potential personal and professional harm caused by disclosure of medical information or personal behaviors [75]. The presence of ethics of care factors within ISC reflects the importance of this perspective when nurses face ethical dilemmas in practice [68]. Given the ethical challenges introduced by ICT in nursing work, nurses do not rely solely on normative ethics; rather, ethics of care is central to their professional identity. Our interview responses clearly illustrate this, showing that ethics of care informs nurses’ reasoning on information security issues. Like normative ISC factors, ethics of care ISC factors reflect a collective, caring norm focused on protecting patient vulnerabilities from information security breaches.

Another reason for the emergence of ethics of care ISC factors may be the limited effectiveness of normative ethics in preventing information security breaches. Blanken‐Webb [55] argues that predetermined principles and laws alone are insufficient for protecting patient privacy and security. Noddings [76] proposes shifting the focus from the potential consequences of breaches to the caregiver’s mindfulness in preventing such events, which is a hallmark of ethics of care. The implementation of the General Data Protection Regulation (GDPR) [73] significantly influences nursing practice, particularly regarding confidentiality, processing, and sharing of patient data. Healthcare professionals bear a crucial responsibility to prevent misuse of personal information by ensuring institutional safeguards and upholding ethical principles. GDPR’s emphasis on good faith and respect for individual rights closely aligns with the nursing ethical code [11], reinforcing the importance of protecting patient privacy during care.

While this study offers valuable insights into ISC in nursing, certain limitations must be acknowledged. First, qualitative research is inherently susceptible to bias due to researchers’ prior knowledge, beliefs, and characteristics [77]. To minimize this, we employed a clear, systematic research protocol following best practices [60, 77], including standardized interviews, avoiding reflexivity, verifying transcripts, and independent coding by two researchers to limit subjective influence. Second, since the sample comprised Slovenian nurses and IT experts, the findings may not generalize to nursing ISC in other cultural contexts. Further research should validate these ISC factors across diverse sociocultural settings. Third, participation was voluntary, possibly attracting individuals interested in information security, which may bias findings. Including participants without prior interest might reveal additional ISC insights. Future studies could explore the relationship between ISC factors and nurses’ actual security behavior, necessitating operationalization of these factors for measurement tools. As ethics extends beyond nursing, the proposed nursing ethics ISC framework may be applicable in other contexts, meaning that ethics of care ISC factors may be relevant in addition to normative ISC factors for professions where these perspectives are fundamental.

## Conflicts of Interest

The authors declare no conflicts of interest.

## Funding

The authors received no financial support for the research, authorship, and/or publication of this article.

## Data Availability

The data that support the findings of this study are available on request from the corresponding author. The data are not publicly available due to privacy or ethical restrictions.
